# An Investigation of the Cytotoxicity and Caspase-Mediated Apoptotic Effect of Green Synthesized Zinc Oxide Nanoparticles Using *Eclipta prostrata* on Human Liver Carcinoma Cells

**DOI:** 10.3390/nano5031317

**Published:** 2015-08-12

**Authors:** Ill-Min Chung, Abdul Abdul Rahuman, Sampath Marimuthu, Arivarasan Vishnu Kirthi, Karunanithi Anbarasan, Govindasamy Rajakumar

**Affiliations:** 1 Department of Applied Bioscience, College of Life and Environmental Science, Konkuk University, Seoul 05029, Korea; E-Mail: imcim@konkuk.ac.kr; 2 Unit of Nanotechnology and Bioactive Natural Products, Post Graduate and Research Department of Zoology, C. Abdul Hakeem College, Melvisharam 632509, Vellore District, Tamil Nadu, India; E-Mails: abdulrahuman6@hotmail.com (A.A.R.); mari.ubanp2009@gmail.com (S.M.); bioviski@gmail.com (A.V.K.); anbuk97@gmail.com (K.A.)

**Keywords:** anticancer actions, caspase study, zinc oxide nanoparticles, *Eclipta prostrata*

## Abstract

Cancer is a leading cause of death worldwide and sustained focus is on the discovery and development of newer and better tolerated anticancer drugs, especially from plants. In the present study, a simple, eco-friendly, and inexpensive approach was followed for the synthesis of zinc oxide nanoparticles (ZnO NPs) using the aqueous leaf extract of *Eclipta prostrata*. The synthesized ZnO NPs were characterized by UV-visible absorption spectroscopy, X-ray diffraction (XRD), Fourier transform infrared spectroscopy (FTIR), Scanning electron microscopy with energy dispersive X-ray spectroscopy (SEM-EDX), High-resolution transmission electron microscopy (HRTEM), and Selected area (electron) diffraction (SAED). The HRTEM images confirmed the presence of triangle, radial, hexagonal, rod, and rectangle, shaped with an average size of 29 ± 1.3 nm. The functional groups for synthesized ZnO NPs were 3852 cm^−1^ for H-H weak peak, 3138 cm^−1^ for aromatic C-H extend, and 1648 cm^−1^ for Aromatic ring stretch. The 3-(4,5-Dimethylthiazol-2-yl)-2,5-Diphenyltetrazolium Bromide (MTT), caspase and DNA fragmentation assays were carried out using various concentrations of ZnO NPs ranging from 1 to 100 mg/mL. The synthesized ZnO NPs showed dose dependent cytopathic effects in the Hep-G2 cell line. At 100 mg/mL concentration, the synthesized ZnO NPs exhibited significant cytotoxic effects and the apoptotic features were confirmed through caspase-3 activation and DNA fragmentation assays.

## 1. Introduction

Cancer is a deadly class of diseases whose mortality levels have increased every year [1]. It is drawn by uncontrolled cell division because of various hereditary modifications happening inside a cell which may modify the harmony in the middle of the multiplication and modified cell demise (apoptosis) component [2,3]. Hepatocellular carcinoma is a standout amongst the most widely recognized growths on the planet and it is a multifactorial disease, caused due to smoking, beverage, poisons, and the human hepatitis disease. Impelling cell apoptosis is a critical method for killing malignancy cells. Apoptosis is a modified cell passing that prompts the end of unwanted, harmed, or tainted cells. Cells undergo apoptosis through distinct pathways, including fatty acid synthase (Fas) and Fas ligand (FasL), which results in the activation of the caspase-8, mitochondria-dependent path and the caspase-3-dependent pathway, triggering the cytoplasmic release of pro-apoptotic mitochondrial pro-apoptotic mitochondrial proteins before leading to apoptosis [4]. To beat this issue, it is important to create and plan new techniques, apparatuses, and drugs for the investigation and treatment of malignancy [5]. Currently, nanoparticles and nanomaterials could be commonly utilized as a part of a choice, which incorporates focus on medication conveyance, imaging, analysis, beautifiers, and biosensors [6].

A variety of manufactured metal oxide nanoparticles are being developed and incorporated into products where their unique catalytic capability, optoelectronic features, antimicrobial activity, and other characteristics make them attractive for a broad series of applications [7]. Zinc oxide (ZnO) nanopowder is at present used in products including plastics, ceramics, glass, reinforcer, rubber, lubricants, paints, pigments, foods (source of Zn nutrient), batteries, and fire retardants [8]. The plant mediate nanoparticle fusion is a cost effective method and therefore a forthcoming commercial alternative for large-scale production [9]. Pharmaceutical nanotechnology, with its numerous advantages, has growingly attracted the attention of many researchers [10]. The old authors reported that the biosynthesis of ZnO NPs using leaf extract of *Calotropis gigantea* [11], *Aloe barbadensis* (*Aloe vera*) [12], and *Corriandrum sativum* [13]), showed antimicrobial activity. *Eclipta prostrata* (Asteraceae) is a widely distributed medicinal plant originating in China, Korea, India, Philippines, and Japan [14]. The plant has a folk reputation in Taiwan as a remedy for the treatment of bleeding, hemoptysis and itching, hepatitis, diphtheria, and diarrhea. The compounds isolated from *E. prostrata* were stigmasterol, caffeic acid, wedelolactone [15], thiophene derivatives, steroids, triterpenes, flavonoids, polyacetylenes, polypeptides, and coumestons [16]. In the present study, biomedical applications of synthesized ZnO NPs using aqueous leaf extract of *E. prostrata* were carried out to assess the anticancer activities (3-(4,5-Dimethylthiazol-2-yl)-2,5-Diphenyltetrazolium Bromide (MTT) assay, Caspase-3, -8, -9 assay, and DNA fragmentation assay) against Hep-G2 cell lines.

## 2. Results and Discussion

### 2.1. UV-Vis Spectroscopy

**Figure 1:**
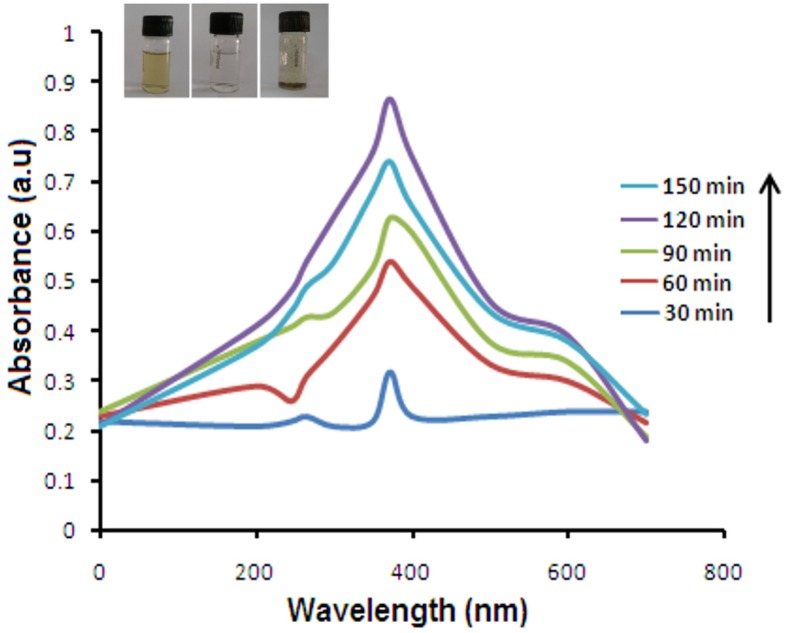
UV-Vis spectral analysis and color intensity of biosynthesized NPs at various time intervals of synthesized ZnO NPs at 120 min. The inset shows the color change from yellow to ruby-red in color.

ZnO NPs had intensive absorption in the ultraviolet band of about 300–700 nm. At a 372 nm wavelength, synthesis was observed for ZnO NPs with optimum temperature of 80 °C and pH of 8.5, with 5 mM Zn (NO_3_)_2_ during a 120 min incubation period. A biosynthesis method was employed for preliminary evaluation of the reducing potential of *E. prostrata* extract by controlling different physico-chemical parameters. Decline of Zn(NO_3_)_2_ was visually evident from the color change and completed within 120 min with a stable ruby-red color indicating the formation of ZnO NPs. UV-Vis study disclosed the fact that the leaf extract of *E. prostrata* exhibited rapid and stable synthesis of ZnO NPs, which showed peak absorption at 372 nm (Figure 1).

### 2.2. X-ray Powder Diffraction Analysis

**Figure 2:**
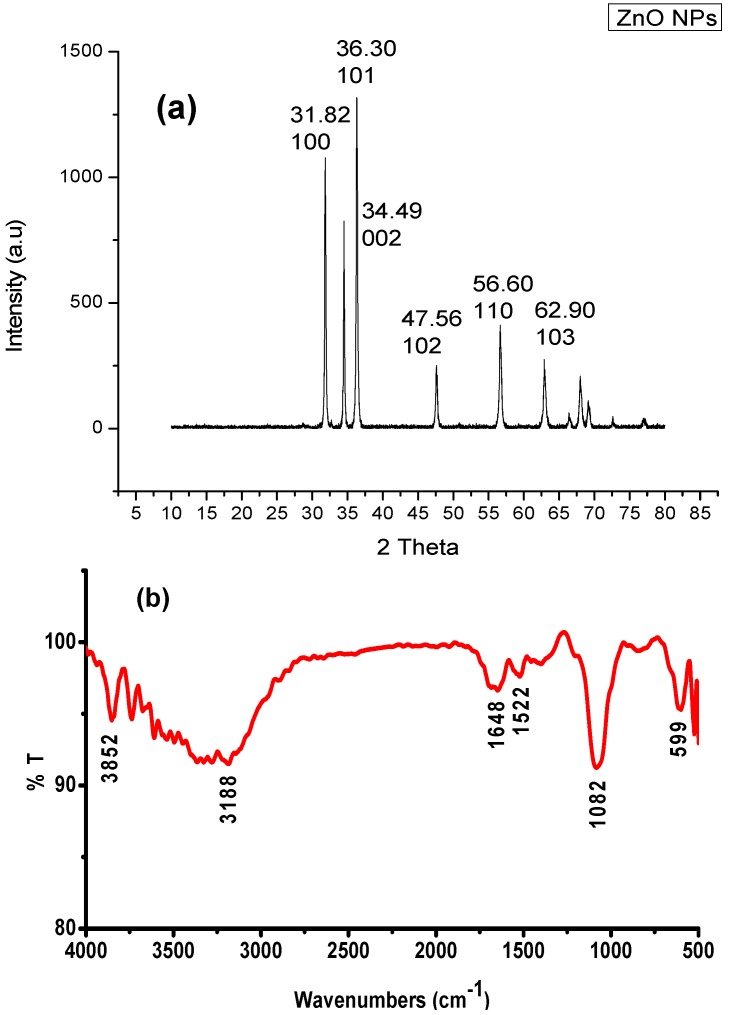
(**a**) X-ray diffraction (XRD) patterns of biosynthesis of ZnO NPs using aqueous extract of *E. prostrate*;(**b**) Fourier transform infrared spectroscopy (FTIR) peaks of synthesized ZnO NPs using *E. prostrata* aqueous leaf extract.

The X-ray diffraction pattern of ZnO NPs synthesized by leaf extract is shown in Figure 2a. The XRD pattern showed four intense peaks in the whole spectrum of 2θ values ranging from 20 to 80. The XRD spectrum compared with the standard confirmed spectrum of zinc particles formed in the present experiments were in the form of nanocrystals, as documented by the peaks at 2θ values of 31.82°, 36.30°, 34.49°, 47.46°, 56.60°, and 62.90° comparative to 100, 101, 002, 102, 110, and 103 planes for zinc, respectively. Similarly, Rajiv *et al.* [17] reported that the peaks were recognized as (100), (002), (101), (102), (110), (112), and (202) reflections, in ZnO NPs synthesized using leaf extract of *Parthenium hysterophorus*. In the present findings, peaks at 2θ values and the corresponding planes confirmed the synthesis of ZnO NPs using leaves of *E. prostrata*.

### 2.3. FTIR Spectroscopy

The FTIR bands of biosynthesized ZnO NPs using leaf extract of *E. prostrata* were indicated at 3852 cm^−1^ (H–H weak peak), 3138 cm^−1^ (Aromatic C–H stretch), 1648 cm^−1^ (Aromatic ring stretch), 1522 cm^−1^ (Aromatic nitro compounds) and 1082 cm^−1^ (organic siloxane or silicone (Si–O–Si) (Figure 2b). The FTIR spectrum of extracellular mycosynthesis of ZnO NPs by *Alternaria alternata* showed the absorption band at 1627 cm^−1^ corresponding to the amide I of polypeptides [18]. The FTIR analysis of synthesis of ZnO NPs using seaweeds showed the bands at 1736 cm^−1^ representing C=O carboxylic acid and the strong C–H group bonds at 1023 cm^−1^, which were sharper and broader for ZnO NPs participates in the reaction [9]. The FTIR peak of synthesized ZnO NPs using *Aeromonas hydrophila* observed the peak at 1635 cm^−1^ (medium charge) vinyl, cis-tri substituted; and 1385 cm^−1^ -monosubstitued alkyne, 1115 cm^−1^ -alkanes, mononuclear benzene ring [19]. The present study revealed that the FTIR band proved the appearance of amide I, carboxylic acid, and strong aromatic ring, which may be responsible for the synthesis of ZnO NPs using leaves of *E. prostrata*.

### 2.4. SEM-EDX Analysis

**Figure 3:**
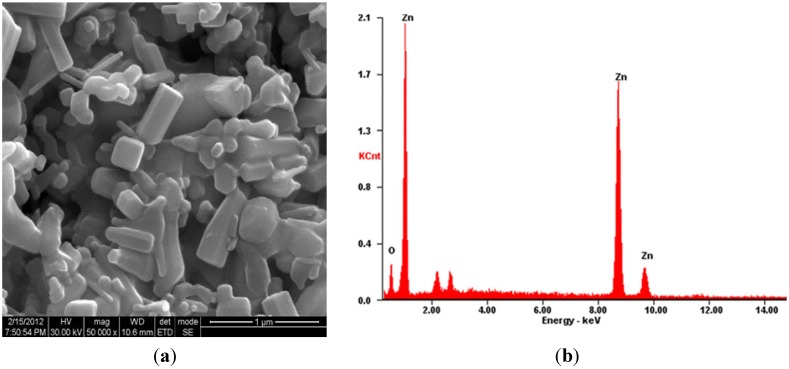
(**a**) SEM micrograph showed the synthesized of ZnO NPs using *E. prostrata* leaf aqueous extract; (**b**) EDX analysis showing the chemical composition of synthesized ZnO NPs.

The scanning electron microscopy (SEM) has been used to examine the surface morphology and to estimate the obtained organic rectangle, triangle, radial hexagonal, rod, and spherical shapes in synthesized ZnO NPs using *E. prostrata* leaf extract (Figure 3a). Further observations exposed that ZnO NPs were in the nanoscale range of 20–75 nm with an average size of 44 ± 0.9 nm. The energy dispersive X-ray spectroscopy (EDX) spectroscopy of synthesized ZnO NPs depicting the chemical components present in the sample identified zinc oxide, and the EDX analysis showed 82% of zinc and 18% of oxides, which confirms the elemental composition of ZnO NPs (Figure 3b). The ZnO NPs synthesized using *Sargassum myriocystem* extract, the surface morphology, and gauging the acquired structural rectangle, triangle, radial hexagonal, rod, and circular shapes, which revealed its size as 96–110 nm by utilizing SEM and the EDX analysis, were 52% zinc and 48% oxides, which confirms the elemental composition of ZnO NPs [20].

### 2.5. HRTEM Analysis and SAED Pattern

**Figure 4:**
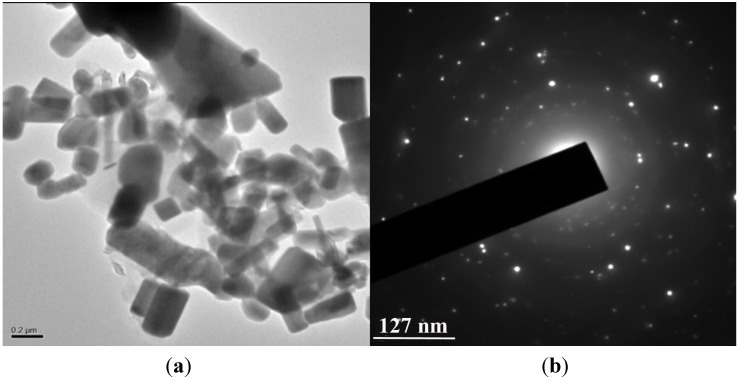
(**a**) High-resolution transmission electron microscopy (HRTEM) image showing synthesized ZnO NPs from *E. prostrata* leaf extract; (**b**) Selected area of electron diffraction pattern (SAED) of the synthesized ZnO NPs showing the rings.

A thin layer of leaf extract of *E. prostrata* was coated with ZnO NPs, using leaf extract as capping agent; a few agglomerated ZnO NPs were also observed in some places. Most of the ZnO NPs fell inside the range of different sizes of triangle, radial, hexagonal, rod, and rectangle atoms with the sizes ranging from 16 to 85 nm and with an average size of 29 ± 1.3 nm (Figure 4a). The Selected area (electron) diffraction (SAED) pattern suggested that the particles were highly crystalline in nature as shown in inset Figure 4b. The organic method for the formation of ZnO NPs utilizing *Calotropis procera* smooth latex at room temperature was reported, and the picture demonstrated the normal size of 5–40 nm and SAED pattern displaying an arrangement of rings containing spots, proposing that nanoparticles have a bigger grain size, uniform shape, and polycrystalline in nature [21].

### 2.6. Anticancer Activity

#### 2.6.1. Cytotoxicity Study of Hep-G2 Cell Line

The cytotoxicity of ZnO NPs in *in vitro* conditions of Hep-G2 cells was examined in terms of effect on cell proliferation by MTT assay for 24 h. After 24 h of post treatment, the samples were tested in various concentrations of 1, 10, 100, 250, and 500 μg/mL, which showed the cell necrosis of 14.5%, 51.5%, 67%, 84%, and 86.5%, for ZnO NPs, respectively, and watched for cytopathic effects, confirming the nature of the NPs to be radical towards the cell lines. A few different studies had reported that cell uptake and genotoxicity of ZnO NPs had likewise been accounted for *in vitro* and *in vivo* [22,23]. The elevated ROS levels induce significant damage to the DNA of the cells, resulting in cell-cycle arrest and subsequently cell death [24]. The p53 can function as an important regulator in determining ZnO induced cytotoxicity, highlighted by the differential action of ZnO on p53 deficient and proficient colorectal cell lines. The p53 deficient cells cancer cells such as DLD-1 and SW480 are more susceptible to ZnO induced cell death compared to p53 proficient cells such as colon epithelial cells NCM460 and HCT116 cells in a ROS dependent manner [25]. In addition, the cell dimensionality plays a critical role in governing the spatiotemporal cellular outcomes like inflammatory response and cytotoxicity in response to ZnO NPs treatment [26]. The dissolution of ZnO nanoparticles and Zn^2+^ release were capable of ROS generation and activation of an integrated cytotoxic pathway that includes intracellular calcium flux, mitochondrial depolarization, and plasma membrane leakage [27]. There are some reports that at the non-cytotoxic ZnO NPs level of 10 mg/L could elevate the intracellular oxidative stress, inducing viability loss, membrane leakage, and morphology changes [28,29,30]. The cellular responses, such as apoptosis in the presence of ZnO nanoparticles, require p53 as the molecular master switch towards programmed cell death and that in cells without robust p53, protective response can be tipped towards carcinogenesis when stimulated by DNA damage-inducing agents like ZnO nanoparticles [31].

#### 2.6.2. Caspase-3, -8, -9 Assays

**Figure 5:**
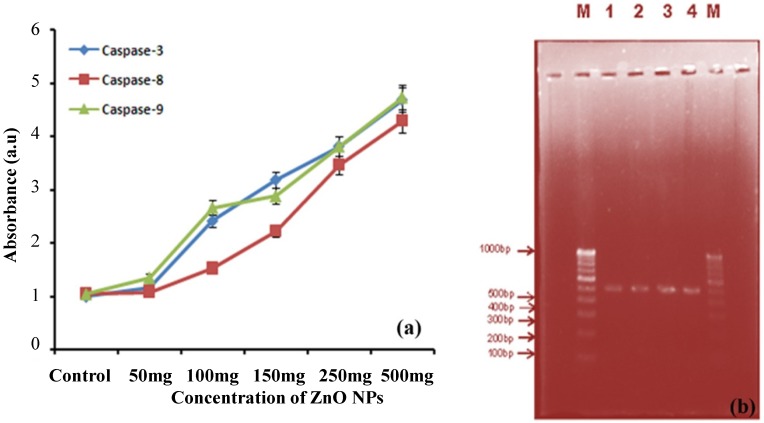
(**a**) Caspase-3, Caspase-8 and Caspase-9 activity of synthesized ZnO NPs; (**b**) DNA fragmentation: M is 100 bp DNA ladder used as a marker DNA; 1-Control (medium); 2-Camptothecin treated; 3-Doxorubicin treated and 4-DNA of ZnO NPs treated cells showing a large DNA ladder indicates more apoptosis.

Caspase-3, -8, -9 activities were measured in Hep-G2 cells treated with 50–500 μg/mL of ZnO NPs for 24 h, resulting as 1.15, 2.41, 3.18, 3.81 and 4.68 a.u.; 1.08, 1.52, 2.21, 3.46 and 4.28 a.u.; and 1.35, 2.66, 2.88, 3.81 and 4.73 a.u., respectively. These results suggest that ZnO NPs can potentially change apoptotic protein expression and trigger apoptosis in mitochondria-dependent pathways in Hep-G2 cells (Figure 5a). A late study on ZnO NPs harmfulness to the Jurkat cell recommended an ionic impact including the extracellular release of high dimensions of Zn, their quick uptake by the cell, and the instigation of a caspase-independent option apoptosis pathway that is autonomous of ROS development [32].

#### 2.6.3. DNA Fragmentation Assay

To investigate whether biologically synthesized nanoparticles induced cell death via apoptosis, DNA laddering assay was done on agarose gel. The results showed that the synthesized ZnO NPs using leaf extract of *E. prostrata* caused internucleosomal DNA fragmentation of 1 kb ladder in the liver cell line exhibiting the characteristic features of apoptosis. The power of fragmented DNA bands was prominent in cells (as shown by agarose gel electrophoresis) treated with *E. prostrata* when compared to control cells (untreated) that showed no fragmentation. There was a smear and faint band of 600 bp in Camptothecin and Doxorubicin treated cells. It was observed that there was an increase in internucleosomal DNA fragmentation due to activation of the intracellular caspase enzyme and oxidative stress in cells in the present study. Therefore, the data obtained from this study confirms that ZnO NPs induced cell death through apoptosis (Figure 5b). DNA ladders of the corresponding treated samples confirmed apoptosis and showed that the Ag NPs-treated DLA cells exhibited extensive double strand breaks, thereby yielding a ladder appearance, while the DNA of control DLA cells supplemented with 10% serum exhibited minimum breakage. The 1 kb ladder was used to find the molecular weight of cleaved DNA fragments [33]. Despite the fact that growth in the comet parameter was watched for the other ZnO NPs concentrations, the change was not noteworthy contrasted with restrain. The genotoxic potential of ZnO NPs (~20 nm) and their ability to discompose the mitochondrial membrane potential, maybe through oxidative stress, in human peripheral blood mononuclear cells has been reported by Javed *et al.* [34].

It was observed that there was an increase in internucleosomal DNA partition due to activation of intracellular caspase enzyme and oxidative stress in cells in the present study. It has been accounted for that the ZnO and ZnPc NPs, which animate the apoptotic signaling pathway and encourage the DNA fragmentation that implies that biochemical signs of apoptosis and grapheme nanoparticles, also incite the oxidative stress in mitochondria and apoptotic DNA fragmentation [35]. Previous cytotoxicity studies on BEAS-2B and RAW264 cells have shown induction of the intracellular Ca^2+^ flux, bringing down of the mitochondrial membrane potential, and loss of membrane uprightness after preclusion to 20 nm ZnO NPs [36].

## 3. Experimental Section

### 3.1. Collection of E. Prostrata

The leaves of *E. prostrata* were collected from Yelagiri Hills (12°34′41″ N, 78°38′27″ E), Vellore district, South India, in January 2010. The taxonomic identification was designed by Chandra Hema, Department of Botany, Arignar Anna Govt. Arts College for Women, Walajapet, Vellore, India. The receipt specimen was numbered (EP/ZD/RK/695/10) and kept in our examination laboratory for further reference.

### 3.2. Preparation of Plant Extract

Fresh and healthy *E. prostrata* leaves were gathered, washed thoroughly with distilled water, broken into small pieces and air-dried. About 10 g of finely cut leaves were weighed and transferred into a 250 mL beaker containing 100 mL of distilled water, mixed well and boiled for 15 min. The extract obtained was drinkable through Whatman No. 1 filter paper and the residue was collected in a 250 mL Erlenmeyer flask and stored in a refrigerator for further use [37].

### 3.3. Biosynthesis of ZnO NPs

The aqueous extract of *E. prostrata* was prepared using freshly collected leaves (10 g). The leaves were surface cleaned with running tap water, in which distilled water is added and boiled with 100 mL of double-distilled water at 120 °C for 1 h. The extract was filtered through Whatman No. 1 filter paper (GE Healthcare Life Sciences, Seoul, Korea) followed by nylon mesh and used for further experiments. For synthesis of ZnO NPs, the Erlenmeyer flask containing 100 mL of zinc nitrate (Zn(NO_3_)_2_)·(5 mM) was stirred for 3 h and 25 mL of the aqueous leaf extract of *E. prostrata* was added to 75 mL of 5 mM Zn(NO_3_)_2_ at room temperature under stirred conditions for 48 h. After the reaction of *E. prostrata* extract with Zn(NO_3_)_2_ the color changed to light green [12]. The ZnO NPs were subjected to vigorous stirring at 150 °C for 5–6 h, allowed to cool at room temperature, and the supernatant was discarded. The pale white solid product obtained was centrifuged twice at 4500 rpm for 15 min after thorough washing, and dried at 80 °C for 7–8 h. The resulting dried precursor was crushed into powder and stored in an airtight container for further analysis.

### 3.4. UV-Vis Spectroscopy

UV-Vis spectral analysis was done by using a Schimadzu 1601 spectrophotometer (Dong-il SHIMADZU Corporation, Seoul, Korea) with a resolution of 1 nm between 200 and 700 nm. The reduction of ions was monitored by measuring the UV-Vis spectrum of the reaction medium after diluting a small aliquot of the sample in deionized water. Using a pipette, 1 mL of the sample was transferred to a test tube and diluted with 4 mL of deionized water and subsequently analyzed at room temperature.

### 3.5. Characterization of ZnO NPs

The dried and powdered synthesized ZnO NPs were used for X-ray diffraction (XRD) spectroscopy (Diffractometer with Philips^®^ PW 1830 X-ray generator, Philips, Amsterdam, The Netherlands). Fourier transform infrared (FTIR) spectrum (Perkin Elmer, Waltham, MA, USA) was measured using a Perkin Elmer Spectrum One instrument in the diffuse reflectance mode. For the scanning electron microscopic (SEM) studies, 25 μL of the sample was sputter-coated on a copper stub, and the pictures of nanoparticles were studied using SEM (ModelJFC-1600, JEOL USA, INC., Peabody, MA, USA). The ZnO NPs for SEM analysis were prepared by evaporating a drop of propan-2-01 solution containing the nanoparticles onto an amorphous carbon film supported on a copper mesh grid.

### 3.6. Anticancer Activity

Energy broadcasted X-ray spectroscopy (EDX) (Oxford Instrument, Oxford, UK) was carried out to determine the chemical composition of the synthesized ZnO NPs. The size and morphology of synthesized ZnO NPs were examined using HRTEM analysis. The samples were mounted on carbon-coated copper grids and the grids were allowed to dry in air prior to measurements on a microscope (JEOL Model 3010) operated at an accelerating voltage of 200 keV with wavelength of 0.0251 Å.

#### 3.6.1. Cytotoxicity Study of Hep-G2 Cell Line

The cytotoxicity effect of synthesized ZnO NPs as well as aqueous leaf extract on Hep-G2 cancer cell line was performed [38]. Hep-G2 cell lines were obtained from the National Centre for Cell Science (NCCS), Pune. Hep-G2 cells were cultured and seeded in 96 well plates with approximately 1 × 10^4^ cells in each plate and incubated for 48 h. The cancer cells were plated separately in 96 well plates at the concentration of 1 × 10^5^ cells/well. Then the cells were treated with different concentrations of ZnO NPs (1–500 μg/mL) and incubated in the presence of 5% CO_2_ and 95% humidity at 37 °C for 24 h. MTT (0.5 mg/mL) was added to the incubated cells, and then they were incubated for another 4 h. The crystals were then dissolved by adding 100 μL of dimethyl sulphoxide (DMSO) and the absorbance was measured in a colorimetric at 570 nm with reference filter as 655 nm.

#### 3.6.2. Caspase -3, -8, -9 Assay

Caspase-3, -8, -9 is a protease known to be involved in apoptotic cell death. HepG2 cells grown to 70%–80% confluency in 12-well plates were incubated for 24 h with various concentrations of phycoerythrin with 10% FBS-DMEM. Approximately 3 × 10^5^ cells were used for ZnO NPs in the concentration at 50, 100 and 150 µg/mL Chromogenic units were converted to percentage by comparing the untreated control cells generated free pNA. The activities of caspase-3 Caspase-8, and Caspase-9 were determined chromogenic by cleavage of substrates, DEVD-7-amino-4-methylcoumarin pNA, VEHD-pNA, and LEHD-pNA, respectively, according to the methods described by Kohler *et al.* [39].

#### 3.6.3. DNA Fragmentation Assay

DNA fragmentation has long been used to distinguish apoptosis from necrosis, and the most reliable methods for detection of apoptotic cells. Then, 1 × 10^6^ Hep G2 cells were plated per well of six-well tissue culture 16 plates and incubated at 37 °C/5% CO_2_ overnight. These cells were treated with 2.0 mL of stock solution suspended in Dulbecco’s modified eagle’s medium (DMEM) containing 10% FBS. Cells were treated with 2 μg/mL Doxorubicin hydrochloride used as controls, along with the ZnO NPs. The cell pellets were treated for 10 s with 50 μL of lysis buffer (1% NP-40 in 20 mM EDTA, 50 mM Tris-HCl, pH 7.5). After centrifugation for 5 min at 1600× *g* (unit rotations per minute) the supernatant was collected and the extraction was repeated with the same amount of lysis buffer. With the supernatant, an additional amount of Sodium dodecyl sulphate (SDS) was added to final concentration of 1% and treated for 2 h with RNase A (final concentration 5 μg/μL) at 56 °C followed by digestion with proteinase K (final concentration 2.5 μg/μL) for 2 h at 37 °C. By the addition of 1/2 μL 10 M ammonium acetate, DNA resulted in precipitated form with 2.5 μL ethanol for obtaining the DNA in a purified form. The DNA pellet was fused in 50 µL of TE buffer, and separated by electrophoresis in 1.0% agarose gel [40]. DNA electrophoresis was performed in 1% agarose gel containing 1 μg /mL ethidium bromide at 70 V, and the DNA segments were visualized by exposing the gel to ultraviolet light, along with photography [41].

#### 3.6.4. Statistical Analysis

All experiments were carried out in triplicate and data were analysed. For the experiments of antimicrobial activity, arithmetic mean values were considered for data analysis. For comparison of the data obtained by the two types of nanoparticles, the unpaired *t*-test was performed. All the statistical analysis was done by SPSS Statistics 18 Release Version 18.0.0, 2009.

## 4. Conclusions

The present biotechnological method capable of producing ZnO NPs at room temperature involving assisted template synthesis of ZnO NPs is a green, high-yield, fast, and low cost approach. The various analyses which followed to characterize the synthesized ZnO NPs revealed that they are triangle, radial, hexagonal, rod, and rectangle in shape with an average size of 29 ± 1.3 nm and crystalline in nature. FTIR spectrum indicated the involvement of different functional activities (amide I, carboxylic acid, and a strong aromatic ring) in the reduction of ZnO NPs. The cytotoxicity data obtained in these results allow us to predict their capability, not only because of the cytotoxic effect, but also in terms of the potential for tumor reduction, which suggests that biologically synthesized ZnO NPs might be used as novel anticancer agents for the treatment of liver cancer.
